# Psychological emotions-based online learning grade prediction *via* BP neural network

**DOI:** 10.3389/fpsyg.2022.981561

**Published:** 2022-09-09

**Authors:** Jiongen Xiao, Hongqing Teng, Han Wang, Jianxing Tan

**Affiliations:** ^1^International Business School of Gdufe, Guangdong University of Finance and Economics, Guangzhou, China; ^2^Electronic Forensics Laboratory, Guangzhou Institute of Software Application Technology, Guangzhou, China; ^3^Department of Law, South China University of Technology, Guangzhou, China; ^4^Department of General and Formative Education, Guangzhou Nanfang College, Guangzhou, China; ^5^Smart City Innovation Center, Fudan Innovation Research Institute, Zhuhai, China

**Keywords:** online learning, psychological emotions, BP neural network, learning status, prediction psychological

## Abstract

With the rapid development of Internet technology and the reform of the education model, online education has been widely recognized and applied. In the process of online learning, various types of browsing behavior characteristic data such as learning engagement and attitude will be generated. These learning behaviors are closely related to academic performance. In-depth exploration of the laws contained in the data can provide teaching assistance for education administrators. In this paper, the random forest algorithm is used to determine the importance of factors for the relationship between 11 learning behavior data and students' psychological quality test data, a total of 12-dimensional feature data and grades, and extracts six factors that have a greater impact on grades. Through the research of this paper, the method of random forest is innovatively used, and it is found that the psychological factor is one of the six important factors. This paper innovatively uses BP neural network as the prediction model, takes six important factors as input, and establishes a complete method of online learning performance prediction. The research in this paper can help teachers monitor students' learning status, detect abnormal learning behaviors and problems in time, and make timely and effective teaching interventions and adjustments in advance according to the abnormal status of students found.

## Introduction

In 1988, Professor Will Smith from Harvard University proposed the concept of online teaching. In 2003, some educational institutions in China gradually began online teaching. In the stage of rapid development of information techniques, online teaching has become a prominent way of learning (Abu Saa et al., 2019). Online learning is well accepted by teachers and students due to its following three characteristics. First, online learning is not affected by the separation between time and space. Second, online learning helps teachers and students make full use of fragmented time. Last but not least, ways of online learning are more flexible. The overall development of online education in China is on the rise since 2016. In 2016, the number of users reaches 104 million, exceeding 100 million for the first time. In 2020, the number of users increases rapidly and reaches 259 million due to the effects of COVID-19, and it was predicted to hike to 446 million in 2021 (Almeda et al., 2018). Many studies indicated that the learning behaviors of learners have a high correlation with learning effects. Traditional face-to-face education utilizes the result-based evaluation method for students. This evaluation method is usually based on the weighted results of usual grades and grades of the final exam, which does not establish close associations between learning behaviors and learning grades of learners. In summary, the result-based evaluation method has one-sidedness to a certain extent, so it fails to fully reflect learners' attitudes and learning habits. Different from traditional face-to-face tutoring, online education can record students' various types of data of learning behaviors in real time to generate learning big data for students. The recorded data are the reflection of students' learning status at that time and their unconscious learning behavior habits formed over a long period of time. A deep study of the learning behaviors helps discover the most authentic learning thinking and learning situations. The activities of comprehensively tracking the learning process of learners, accurately grasping the learning status, and evaluating the learning effects in actual teaching scenarios help teachers take effective measures according to students' status and abilities to realize early identification, early prevention, early intervention, timely, and effective teaching adjustment, so as to greatly improve the teaching quality and students' learning effects.

With the wide application of online education, more and more teachers and students participate, resulting in the generation of great amount of various types of data on platforms. The generated data reflect students' learning behaviors. It is a hot spot to fully mine the patterns hidden in data and to transform structured data as well as unstructured data into effective information. Data mining is applied to the domain of online education by more and more researchers (Chen et al., 2021) to mine the online learning behaviors as well as the correlation between behavior patterns hidden in data and learning effects, to understand the learning behaviors of learners, and to predict the levels of learners to master their knowledge (Elena et al., 2018). However, the prediction for learners' learning grades is complex. The reasons are illustrated as follows. First, learning grades are usually affected by various impact factors such as learners' backgrounds, previous learning performance, the interaction between learners and teachers, and psychological characteristics of learners. Moreover, the types and levels of impact factors are different in different situations (Guo and Liu, 2018). With the help of the research on the impacts of behavior data of learners on learning effects, teachers can monitor the learning status of learners to discover unusual learning behaviors and problems, in order to make timely and effective teaching interventions and adjustments in advance. This study aims to improve the quality of online teaching by predicting students' learning grades, raising students' learning grades, and strengthening online teaching management. The random forest algorithm is applied to recognize important features from various recorded behavior data in platforms. The BP neural network is applied to build a model of online learning behavior and effects according to the selected important behavior data. The built model can predict grades according to learning behavior data during the learning process to monitor learning behaviors, which helps to guide students to carry out beneficial learning behaviors and further improve teaching quality.

## Related work

Online learning behaviors are closely related to learning grades. Learning behavior is an important representation of predicting learning performance. In order to study the impacts of online learning behaviors of learners on their learning grades, the related researches mainly focus on the following categories.

### Many studies on impact factors of learning grades

During the process of online education, large quantities of behavior data are recorded by platforms. Some data have a high correlation with learning grades, while some data have a low correlation. Many researchers have conducted a large number of studies (Abdi, 2010). Shi and Ge (2018) pointed that learning investment and interaction behavior had impacts on grades by reviewing current papers. The authors applied the structural equation model to study the relationship between learning investment (interaction behavior) and grades. Researchers selected behavior data based on previous research findings, which shows subjectivity to a certain extent. Moreover, researchers ignore the impacts of other factors and do not make full use of behavior data recorded by platforms. Zheng et al. (2020) utilized data from the online learning management system (LMS), grades of the final exam, level factors of teachers and classes to study the relationship between learners' activities (teachers' features, class design elements, etc.), and online learning grades. The results indicated that the learning effects will be better when students study courses. Moreover, more times to login and a longer duration of login also lead to better learning effects. Sun and Feng (2019) built models by using neural network, decision tree, and linear regression to study impact factors of online learning grades. It is indicated that learning attitudes (number of subjective and objective questions completed), timely level (the first time to learn courses, etc.) as well as investment (average video viewing progress and times) are the main factors that affected learning achievements. Moreover, the level of frustration tolerance (persistence) has a second-level influence. In addition, the level of interaction (number of posts, replies, and followers), the level of positivity (aggressiveness, etc.), and the stage effect (quality of subjective question completion) showed no correlation with learning grades. Li et al. (2016) used multiple linear regression to analyze 21 online learning behavior indexes. The result indicated that four indexes of the number of assignments submitted, the total number of times when the time interval between each submission of assignments was less than the average time interval of the whole class, the contribution rate of the number of posts, and the average number of browse forum topic posts logged in each time were significantly helpful for predicting grades of courses. Guo and Liu (2018) studied the correlation between seven aspects of online learning behaviors (usual grades, online learning time, online test, forum activities, etc.) and learning effects. These researchers have made full use of online behavior data recorded on platforms to analyze impact levels of behavior data. However, they only use data in platforms, without considering the impacts of individual conditions such as psychological quality on grades.

### Research on relationship between impact factors of learning and grades

With the wide and deep application of information techniques, more and more researchers begin to study the relationship between online learning behaviors of learners and learning grades. Shen et al. (2020) used online learning behavior data to build online learning behavior and learning evaluation model for MOOCAP by using various methods, namely, Delphi, expert ranking, and expert. Shen et al. (2019) used sampling stepwise regression to build the evaluation model for online learning behavior and performance. The impacts of online learning behaviors on learning grades were analyzed. Zhao et al. (2017) utilized multiple regression analysis to determine early warning factors influencing the learning performance of students. The Delphi method is a kind of subjective scoring method. The effectiveness of this method depends on the experts' familiarity with the target industry. The applicability of the regression analysis method is limited because the model structure needs to be estimated first. In addition, the model is also limited by the diversity of factors and the unpredictability of some factors. Sun and Feng (2019) applied neural network, decision tree, and linear regression to build models and recognized the importance of online learning behaviors for learning grades. The authors conducted a quantitative study on the relationship between learning behaviors and learning grades.

### Many studies on models building for learning grade prediction

With the wide application of big data, learning grade prediction has become the important content of data mining for online education. The prediction model is built by discovering the relationship between learning behavior features and grades. The built model can be used to predict the learning grades of learners and further provide an important basis for academic warning, teaching strategy adjustment, and learning plan formulation. Section “Research on relationship between impact factors of learning and grades” recognizes important impact factors but does not validate these factors and applied them to practical scenarios. Song et al. (2020) construct an academic warning model based on the RBF neural network which was optimized by the genetic algorithm. AHP is used to analyze the weights of the impact factors affecting the learning crisis extracted by teachers and experts. The main impact factors are modified according to the weights and then used as the model input for academic early warning. In this case, the recognized important factors are applied to practical scenarios. In the actual process of building models, the redundant or irrelevant features should be reduced without significantly affecting the performance of the model (training time, complexity, and accuracy) (Sun and Feng, 2019). Methods such as AHP, PCA, and methods that select important features by analyzing the influence of different correlations and features on the tags of datasets can be used to reduce the dimensions of model inputs (Tsang et al., 2021). However, AHP and PCA have the shortcoming of strong subjectivity, which is closely related to the familiarity of teachers and experts in this field. As a highly flexible machine learning, the random forest has good accuracy and generates grades for the important attributes during the process of analyzing data. Moreover, unlike algorithms such as SVM, random forest does not need to perform super parameter tuning. The random forest algorithm shows its progressiveness and has been widely applied to practical scenarios.

## Data and methods

### Research object

The Blackboard platform is the online education platform that is currently used in a university in Guangzhou. This platform will store records of users during the process of teaching and learning, namely, course learning statistics records, students' learning records, and teachers' records of accessing and using the platform. Totally 117 courses are provided for students on the platform. The course “Research on mobile applications” in the platform is selected for the research in this paper. In the second semester of the 2019–2020 academic year (March–June 2020), 137 students registered for the study. Totally 11 dimensions of online learning data, including data on average online time backstage, are collected. Except for the online data, the offline grades of the final exam after learning are also included. This paper considers the influences of both online learning behaviors and students' psychological quality which is evaluated by using a questionnaire survey method.

### Data processing and analysis

Indexes of 11 dimensions are achieved by transforming the extracted data from the platform. The indexes are shown in Table 1. The selected course is registered by 137 students. Four students do not participate in the course after registration, so their behavior data and grades are both zero. The sample data of the four students are removed. Moreover, sample data of another student are also removed because the grade of this student is lower than 20 and far lower than the average grades of students in his/her class. This student can answer some subjective questions correctly according to their original knowledge level or by luck to get such a grade even if he/she does not attend the course. The processed data of 132 students are divided into a training set and testing set. The training set is used to establish the prediction model, and the testing set is used to evaluate the prediction accuracy of the model.

**Table 1:** Factors of learning behaviors.

**Category of online learning behavior**	**Typical behavior**
Clicking feature	X1: Number of visits during current semester
	X2: Number of visited pages during current semester
	X7: Number of times to click announcements
	X8: Number of times to click tasks
	X9: Number of times to click “my grades”
	X10: Number of times to click general review questions
	X11: Number of times to click review courseware
Homework test	X3: Number of homework participated during current semester
	X4: Number of tests participated during current semester
Interaction feature (learning emotion)	X5: Number of self-assessment and mutual assessment homework participated
	X6: Number posts during current week
Psychological quality	X12: Psychological quality of a student

Good mental health is conducive to the development of learning potential. This paper comprehensively considers the influences of both online learning features and psychological quality of students on learning grades. The questionnaire for testing students' psychological quality is the college students' psychological quality questionnaire (simplified version) designed by Zhang and Zhang (2018). The questionnaire contains three factors, namely, cognition, personality, and adaptability, and 27 issues. The three factors have nine issues. The questionnaire adopts 5-level scoring. The grades of 1–5 points represent the opinions from “very inconsistent” to “very consistent.” Higher grades indicate a better psychological quality of the tested students. Totally 137 questionnaires were distributed, and 137 valid questionnaires were recovered to obtain the students' psychological quality data. The data are obtained by adding the grades of each issue in the questionnaire and are represented as X12 in Table 1.

The main impact factors are analyzed as follows.

- **Number of visits during the current semester (X1):** The total number of times that a student visits an online platform during the current semester. This index is a reflection of online learning habits and basic learning attitudes. A large number of visits indicates high acceptance of students on the way online teaching and reflects that a student is active in learning and is able to study and review on time.
- **Number of visited pages during the current semester (X2):** The total number of times that a student visits course pages and course-related pages during the current semester. A large number of visited pages indicates a high learning investment (Zhao et al., 2017) of a student and reflects that the student is active in learning.
- **Number of homework participated during the current semester (X3):** This index is the total number of homework that is arranged for a student by teachers during the current semester. This index reflects a student's basic learning attitudes, and the status that the student participates in online learning on time and masters the course progress and content from time to time. At the same time, the student is able to timely consolidate the content of the course through homework.
- **Number of tests participated during the current semester (X4):** This index is the reflection of the basic learning attitudes of students and the total number of tests that are arranged for a student by teachers. The tests are helpful for students to find out his/her weakness in mastering knowledge check the leak and fill the vacancy in the his/her knowledge system.
- **Number of self-assessment and mutual assessment homework participated (X5):** Subjective questions for a student from most online courses can be self-assessed or assessed by other students. The number of self-assessment and mutual assessment homework participants reflects a student's learning enthusiasm. The process of assessing homework is the process of consolidating and further understanding knowledge.
- **Number of posts during the current week (X6):** The number of posts that a student sends during the process of learning a course reflects his/her active participation in the interaction. A large number of posts during the current week indicates a high level of a student's thinking and interactive initiative. This index reflects whether the student thinks seriously in the learning process, whether he/she can actively request help for encountered problems and solve the problems in time.
- **Number of times to click announcements (X7):** This index is the reflection of a student's online learning habits. Generally speaking, a student with good online learning habits will actively check announcements, and he/she will manage to master related activities of courses and complete related tasks for activities in time.
- **Number of times to click tasks (X8):** This index is the reflection of a student's online learning habits. A student with good learning habits usually checks task notifications carefully and timely and completes tasks according to corresponding notifications. On the contrary, a student who seldom pays attention to task notifications is unable to complete tasks arranged by teachers and does not attach importance to learning.
- **Number of times to click “my grades” (X9):** Total number of times that a student checks his/her grades on tests and the final exam. This index reflects the student's attention to learning and his/her sense of honor in learning.
- **Number of times to click general review questions (X10):** This index reflects a student's learning habits of reviewing before an exam, and the time invested by the student for an exam. A larger number of times to click general review questions generally indicates better grades the student will receive.
- **Number of times to click review courseware (X11):** This index reflects a student's learning habits of reviewing after class, and the time spent by the student learning a course after class. A large number of times to click review courseware indicates better grades the student will receive.
- **Psychological quality of a student (Zheng et al.**, **2020****) (X12):** Good mental health is conducive to the development of learning potential. A student with good psychological quality has positive learning attitudes. He/she is optimistic and confident and good at affirming himself/herself. Moreover, he/she will seek help from teachers and other students to solve problems and is good at relieving anxiety. Good psychological quality helps a student to raise his/her learning grades. A student with poor psychological quality may abandon himself/herself due to criticism and dissatisfaction from teachers or parents and lose learning interests. Worse still, he/she may lose confidence or even give up education. A student with good psychological quality can adjust his/her mentality in time when he/she encounters difficult test questions and uses reasonable answer strategies to make full use of his/her knowledge to the greatest extent. On the contrary, a student with poor psychological quality may fall into anxiety because of a certain problem and cannot adjust his/her state in time, which will affect the performance of dealing with subsequent problems, and will not fully reflect his/her ability, so that his/her learning grades are seriously affected.

### Random forest and behavior feature selection

Random forest is an ensemble learning algorithm and it is developed by using the decision tree as a base classifier. The basic idea of random forest is to train multiple decision trees by using data subsets to achieve the voted classification result. Random sampling with replacement is conducted repeatedly to generate K training sample sets N. Each training sample subset corresponds to a decision tree. This process is called the bagging method. The K decision trees created by the bagging method are different from each other, which decreases the correlation between decision trees in random forest and further decreases the error rates of random forest. In random forest, the number of decision trees K and the characteristics of randomly selected node splitting determine the prediction performance of the model. It is one of the characteristics of random forest to estimate the importance of variables. This paper calculates the importance of variables based on the out-of-bag error rate (OOB error). Definition of OOB error estimates (Mitchell, 2011): in random forests, there is no need for cross-validation or a separate test set to get an unbiased estimate of the test set error. It is estimated internally, during the run, as follows: each tree is constructed using a different bootstrap sample from the original data. About one-third of the cases are left out of the bootstrap sample and not used in the construction of the *k*th tree. Put each case left out in the construction of the *k*th tree down the *k*th tree to get a classification. In this way, a test set classification is obtained for each case in about one-third of the trees. At the end of the run, take *j* to be the class that got most of the votes every time case *n* is OOB. The proportion of times that *j* is not equal to the true class of *n* averaged over all cases is the OOB error estimate. This has proven to be unbiased in many tests.

If *x*_*j*_(*j* = 1, 2, ⋯ , 12) is the input variable, then the importance of the *k*th tree *I*_*k*_(*j* = 1, 2, ⋯ , 12) is the mean of estimated error of out-of-bag data before and after randomly replacing variables. The importance is given by Formula (1).


(1) $$\begin{array}{l} I_{k}\left(x_{j}\right)=\left\{\sum_{n=1}^{N_{OOB}}I\left[f\left(x_{n}\right)=f_{k}\left(x_{n}\right)\right]-\sum_{n=1}^{N_{OOB}}I\left[f\left(x_{n}\right)=f_{k}\left({x^{\prime }}_{n}\right)\right]\right\}/\\ \;N_{OOB} \end{array}$$

The importance of variable *x*_*j*_ in random forest is given by Formula (2).


(2) $$\begin{array}{l} I\left(x_{j}\right)=\sum_{k=1}^{K}I_{k}\left(x_{j}\right)/K \end{array}$$

*N*_*OOB*_ is the number of out-of-bag sample data. *f*(*x*_*n*_) is the *n*th sample value of out-of-bag data. *f*_*k*_(*x*_*n*_) and $f_{k}\left({x^{\prime }}_{n}\right)$ are the estimated value of the *n*th sample before and after replacing variables. *I*(·) is the discriminant function whose value is set as 1 when the condition in parentheses is valid and 0 otherwise.

### BP neural network and prediction model design

**Figure 1:**
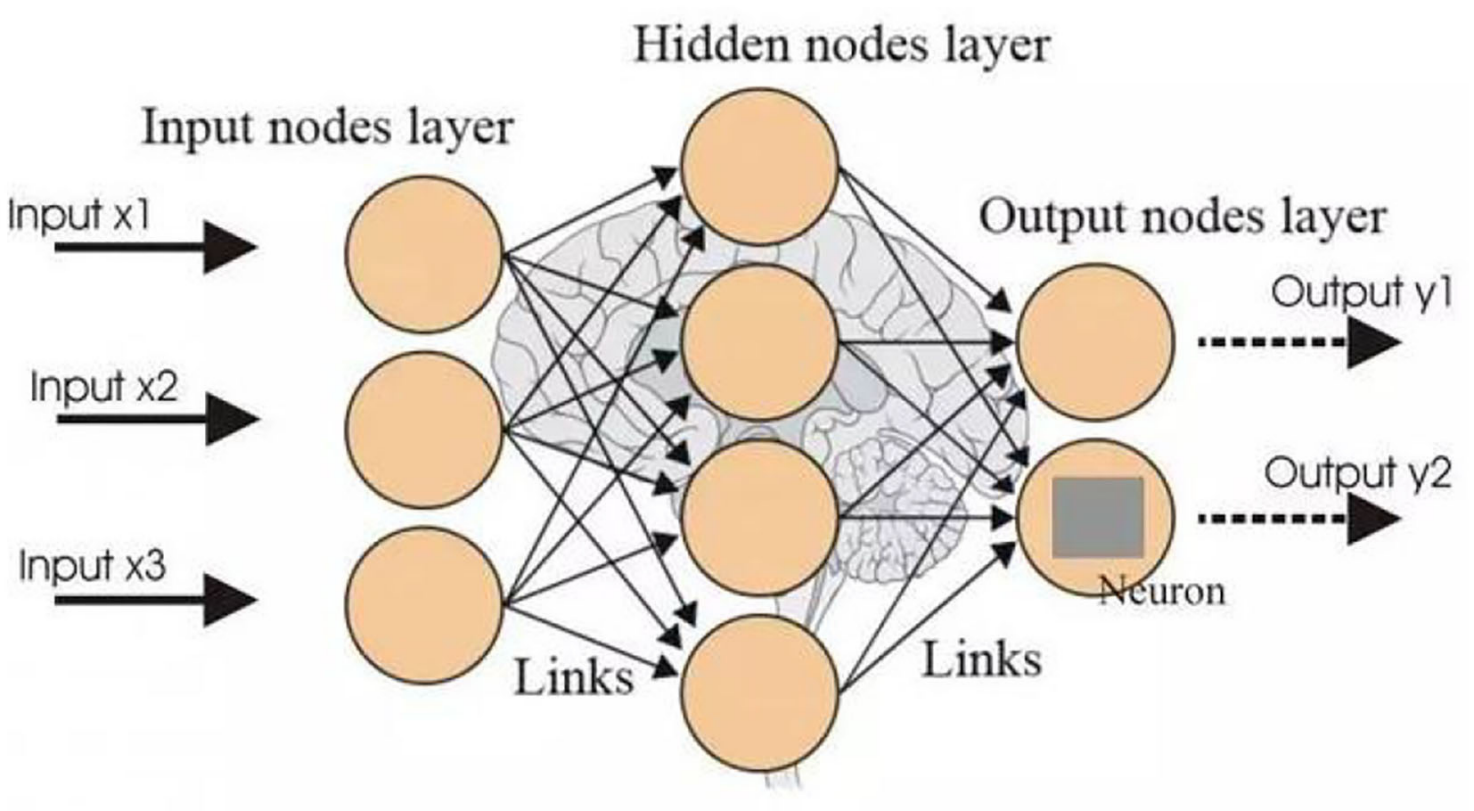
BP neural network structure.

BP neural network (Ding et al., 2011) is one of the most widely used neural network models. It can learn and store a large number of input–output pattern mapping relationships without revealing the mathematical equations to this mapping relationship in advance. It is outstanding in describing non-linear function relationships. A three-layer BP neural network can describe non-linear relationships of any complexity. Its structure is shown in Figure 1.

The selected important behavior feature data of students in Section “Random forest and behavior feature selection” are utilized as the input of the neural network to build a model and conduct prediction. Sigmoid function is selected as the activation function for hidden layers. The parameter setting of the BP neural network is to carry out multiple rounds of tests according to the initial value, continuously optimize to ensure the minimum error, and determine the parameter value through the iterative operation. The number of nodes is set as 7. The output layer generates the grades of students. In this paper, the parameter threshold of the BP neural network model is set as 0.001, the maximum number of training times is set as 6,000, and the learning rate is set as 0.05. When the calculation error is lower than the threshold or the number of training times exceeds the set maximum number, the training is terminated.

## Results and analysis

This paper starts with the relationship between learning behaviors and learning grades to investigate features that influence the learning grades of students. The dimensions of behavior data influencing students' grades are reduced to several typical factors which are helpful to improve students' grade prediction by analyzing the importance of behavior data to retain useful features and remove redundant features. The important behavior feature data are selected as the input to construct BP neural network for students' grade prediction. Based on the aforementioned model design, online behavior data, data of psychological quality, and data of grades of the course “Research on mobile applications” which were offered at the beginning of the semester and learned by 137 students are collected to construct the dataset. The dataset contains valid data from 132 students. About 70% of the dataset is used as a training set, while the other 30% is used as a testing set for evaluating the trained model. The software MATLAB is used as a tool to build the model. This paper is based on the modeling method of random forest. The introduction of random forest (Shi and Horvath, 2006) is as follows.

Random forest is built a forest in a random way. There are many decision trees in the forest, and each decision tree in the random forest is not related. After getting the forest, when a new input sample enters, let each decision tree in the forest make a judgment to see which class the sample should belong to, and then see which class is selected the most, it is predicted that the sample belongs to that class.

A decision tree (Patel and Prajapati, 2018) is a tree structure (can be binary or non-binary). Each non-leaf node represents a test on a feature attribute, each branch represents the output of the feature attribute on a certain value range, and each leaf node stores a category. The process of using a decision tree to make decisions is to start from the root node, test the corresponding feature attributes in the items to be classified, and select the output branch according to its value until it reaches the leaf node, and the category stored in the leaf node is used as the decision result. The analysis of the data in this paper is as follows.

**Figure 2:**
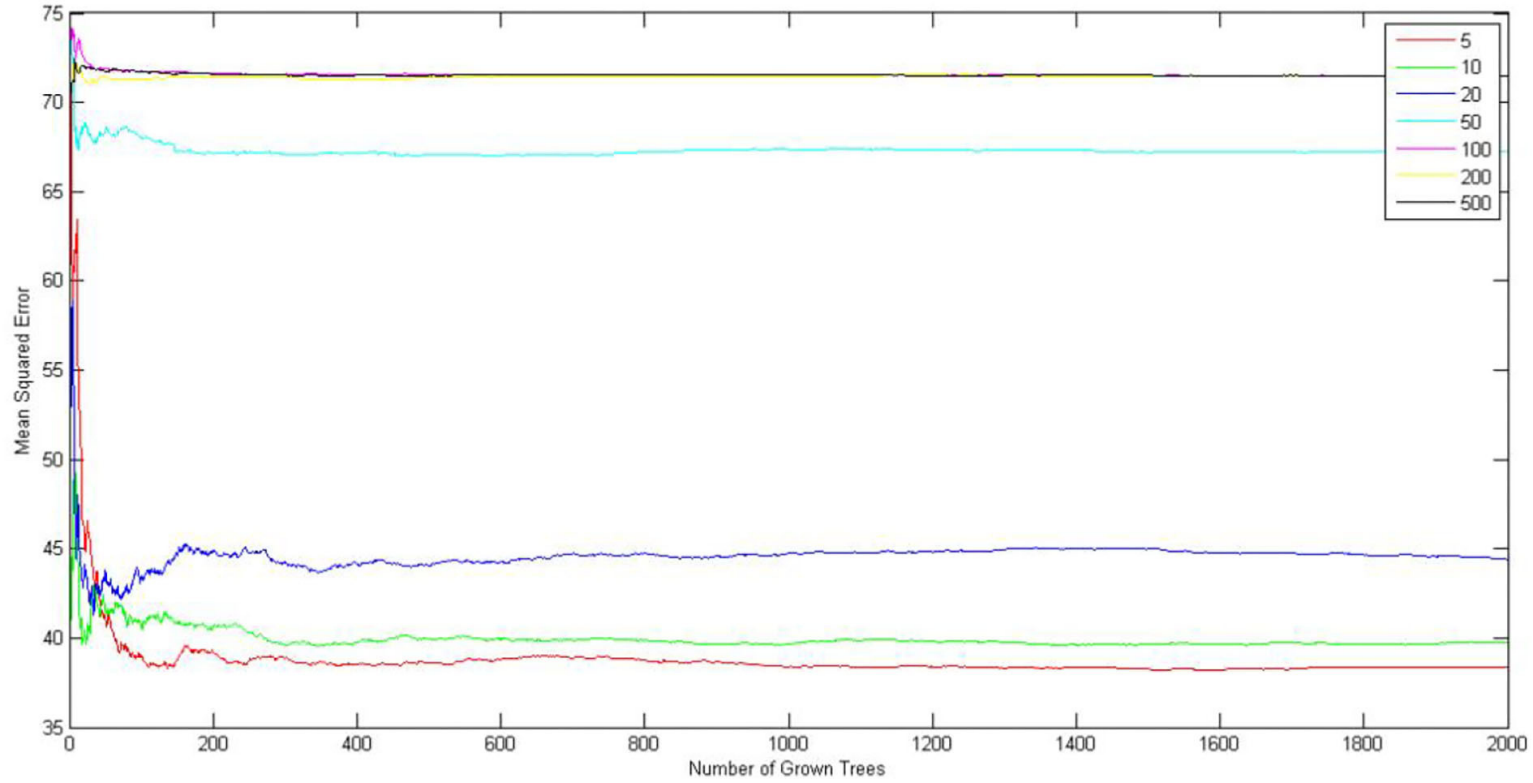
Curve of optimal numbers of leaves and trees in random forest.

First, initial numbers of leaves fall in the range (5, 10, 20, 50, 100, 200, 500). The 132 original data of students are used to select the optimal numbers of leaves and trees. The result is shown in Figure 2. It is indicated that the mean square error of random forest reaches a minimum value when the number of leaves is set as 5, so the optimal number of leaves is 5. By observing the abscissa, it is found that the number of leaves decreases steadily when the number of trees K is 400, so the optimal number of trees is 400.

**Figure 3:**
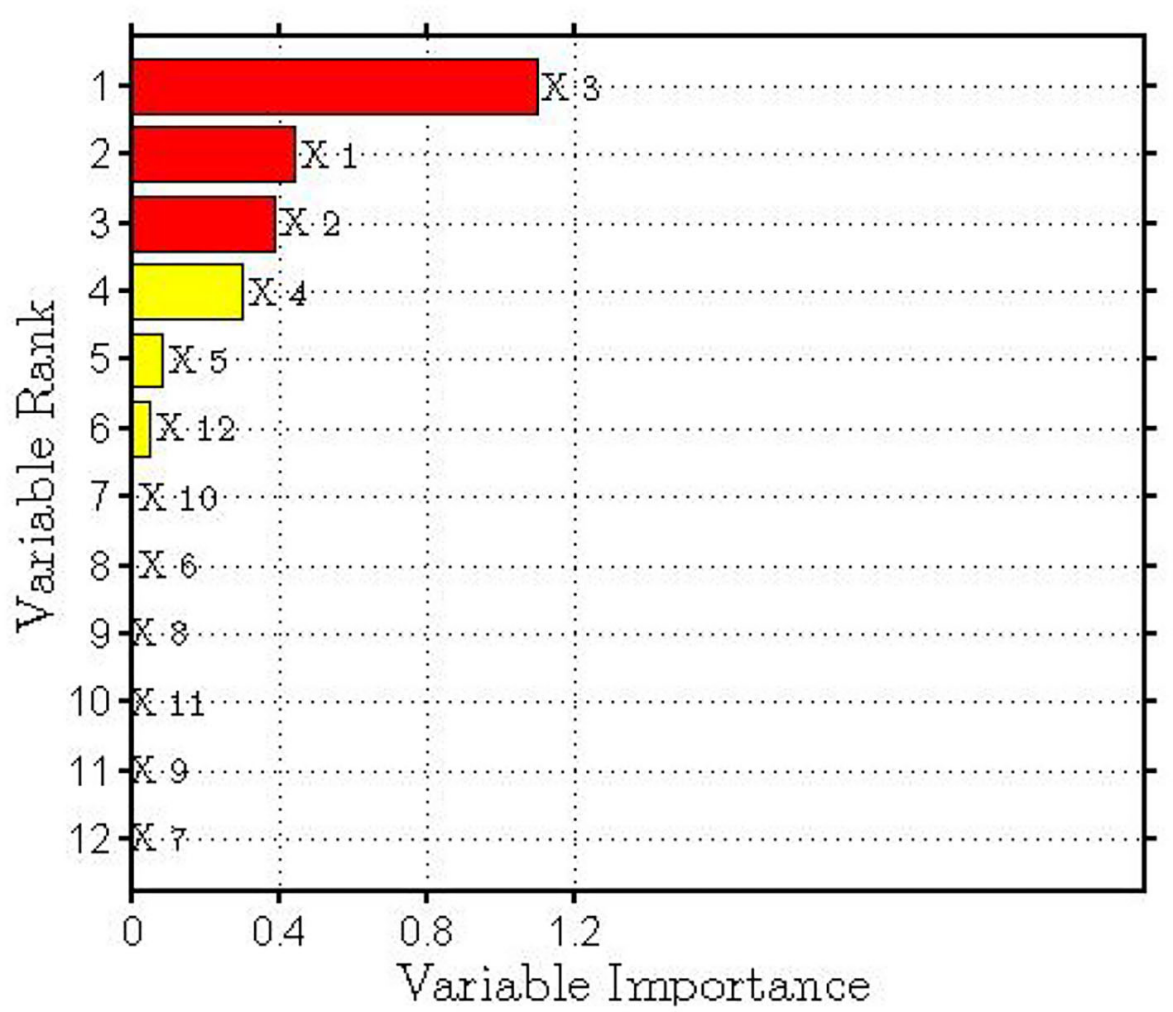
The importance of online behavior features.

Behavior data and psychological quality data of 132 students after the online learning semester are collected to build random forest model with optimal numbers of leaves and trees and the importance of learning behavior features for grades is achieved. The features are sorted and highlighted according to their importance in Figure 3. It can be observed that a total of six features have a significant correlation with learning grades, while the other six features show an insignificant correlation. The six key behavior data, that is, number of visits during current semester (X1), number of visited pages during current semester (X2), number of homework participated during current semester (X3), number of tests participated during current semester (X4), number of self-assessment and mutual assessment homework participated (X5), and psychological quality of a student (X12) are the optimal feature variables for predicting students' grades. The more frequent appearance of these behavior features, the more energy and efforts are invested by students to achieve learning goals and better achievements. According to the above discussion, the six important behavior features are selected as input for BP neural network to train the model and predict students' grades.

**Figure 4:**
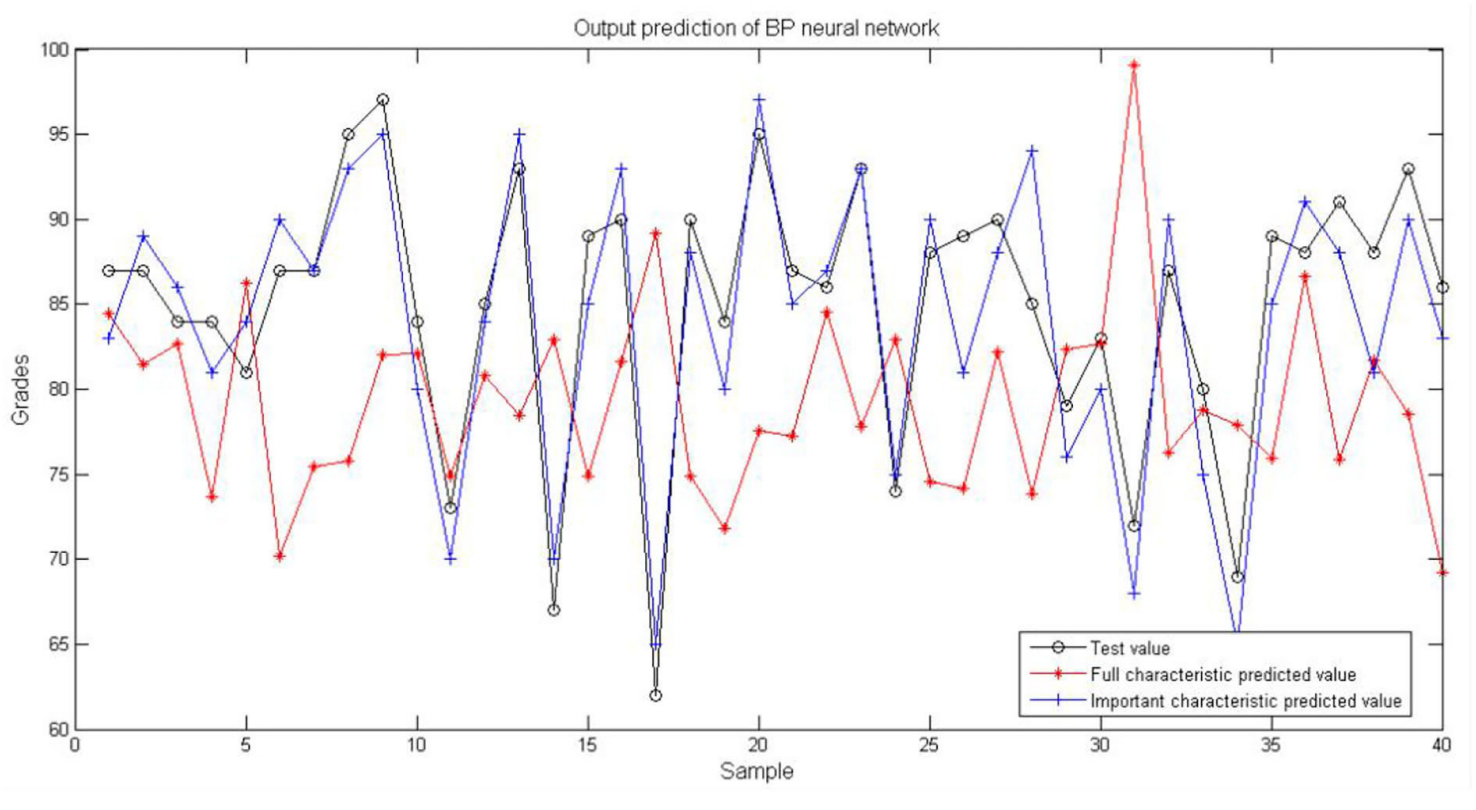
Prediction result of BP neural network.

The recognized six important behavior features are utilized to build the grade prediction model. The 30% (around 40 groups) of the original data are used for the model test. The result is shown in Figure 4. The black curve shows the test sample data. The red curve is the prediction curve using the data of 12 features. The blue curve is the test curve using the important features as input. Obviously, the blue curve achieves better prediction performance because its overall trend is basically consistent with the original sample data. However, the red curve achieves poor prediction performance.

It is indicated in Table 2 that the mean square error of the BP model which is constructed by using important feature data (important characteristic model) is only 3.5468, while the mean square error of the BP model which is constructed by using all the features (full characteristic model) is 14.1968. The former decreases by 75% compared with the latter. For training speed, the training time of the important characteristic model is 0.036974, while the training time of the full characteristic model is 0.060903. The former decreases by nearly half compared with the letter. The comparison of training speed indicates great improvement in the training speed of the prediction model which is constructed using important feature data. The improvement will be more significant in the case of large quantities of data, which greatly reduces computing expenses.

**Table 2:** Comparison of two prediction models.

**Prediction model**	**Maximum prediction error**	**Minimum prediction error**	**Mean square error of predicted value**	**Training time**
Full characteristic model	−32.1144	−0.1412	14.1928	0.060903 second
Important characteristic model	−9.1750	−0.0419	3.5468	0.036974 second

**Figure 5:**
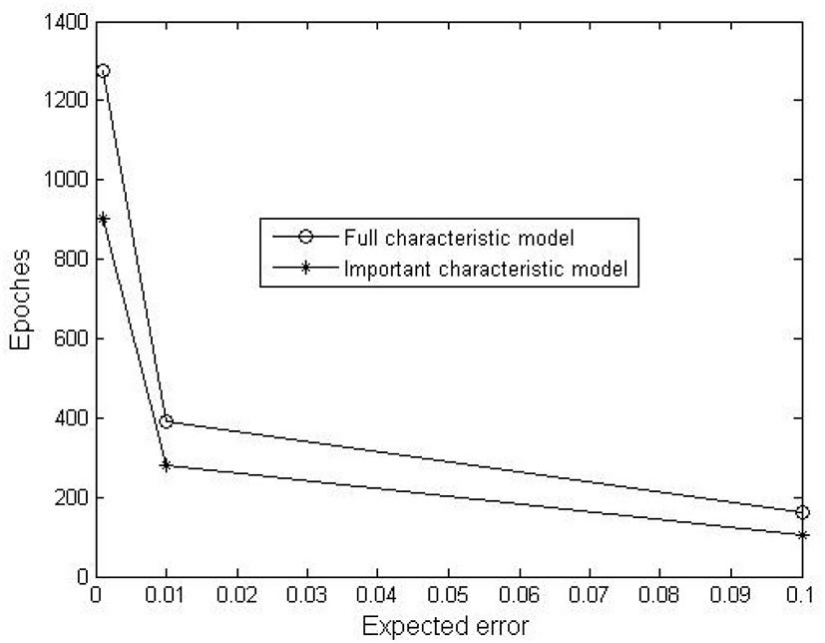
Training effects of two prediction models.

In order to validate the running effects of model, full characteristic model and important characteristic model are trained under the condition that training errors are set as 0.1, 0.01 and 0.001, while other parameters are consistent. The performance of models is shown in Figure 5. It can be observed that the number of iterations of the important characteristic model is obviously smaller, indicating that the training speed becomes faster after the redundant behavior features are removed.

## Conclusion

Based on previous scholars' theories, this paper selects 12 factors that affect students' online learning behavior for research. Using the random forest identification method, six important learning behaviors with significant correlations between students' performance were selected for in-depth analysis. The six important learning behaviors are used as the inputs for BP neural network to build the grade prediction model. The built model removes redundant features, which increases the speed of algorithm, and effectively improves the training speed of network and prediction accuracy.

This paper compares the full feature prediction model and the important feature prediction model. The empirical study also shows that the prediction error of the model established by using the important features is greatly reduced, and the training speed is greatly improved. The model can be used in actual teaching management. Teachers can monitor students' learning behaviors, find abnormal students' behaviors, and conduct teaching interventions in a timely manner, thereby improving the quality of teaching and the effectiveness of students' learning.

## Data availability statement

The original contributions presented in the study are included in the article/supplementary material, further inquiries can be directed to the corresponding author.

## Ethics statement

The studies involving human participants were reviewed and approved by Department of General and Formative Education, Guangzhou Nanfang College. The patients/participants provided their written informed consent to participate in this study.

## Author contributions

JX responsible for writing manuscripts, collecting, and analyzing experimental data. HT responsible for language polishing of articles and emotional analysis of learning behavior. HW responsible for the progress supervision and technical guidance of the overall article. JT responsible for the collection and analysis of data. All authors contributed to the article and approved the submitted version.

## Funding

This research was supported by the Project of Guangdong Science and Technology Department (CN), Grant No. 2020B010166005, the Foshan Social Science Project (2022-GJ048), the Fund project of Department of Science and Technology of Guangdong Province (GDKTP2021032500), and the Post-Doctoral Research Project (Z000158).

## Conflict of interest

The authors declare that the research was conducted in the absence of any commercial or financial relationships that could be construed as a potential conflict of interest.

## Publisher's note

All claims expressed in this article are solely those of the authors and do not necessarily represent those of their affiliated organizations, or those of the publisher, the editors and the reviewers. Any product that may be evaluated in this article, or claim that may be made by its manufacturer, is not guaranteed or endorsed by the publisher.
